# Juvenile Hormone Is an Important Factor in Regulating *Aspongopus chinensis* Dallas Diapause

**DOI:** 10.3389/fphys.2022.873580

**Published:** 2022-05-09

**Authors:** Wen-Zhen Zhou, You-Fang Wu, Zhi-Yong Yin, Jian-Jun Guo, Hai-Yin Li

**Affiliations:** Guizhou Provincial Key Laboratory for Agricultural Pest Management of the Mountainous Region, Institute of Entomology, Guizhou University, Guiyang, China

**Keywords:** *Aspongopus chinensis*, JH, reproduction system, JHEH, JHAMT, diapause

## Abstract

*Aspongopus* *chinensis* is a Chinese traditional edible and medicinal insect, which is in great demand in the society. This insect reproduces once a year which is caused by reproductive diapause resulting in insufficient production in wild resources. However, the mechanism of diapause in *A. chinensis* is still unclear. In this study, we focus on the relationship between juvenile hormones (JHs) and *A. chinensis* diapause. The results showed that JHIII concentration in diapause adult individuals was significantly lower than that in diapause termination adult individuals. When exogenous JHⅢ was injected into diapause adults, the rate of mating was increased significantly, development of the reproductive systems was accelerated, consumption of fat intensified, the expression of juvenile hormone acid o-methyl-transferase (JHAMT) was upregulated, and juvenile hormone epoxide hydrolase (JHEH) and fatty acid synthase (FAS) gene expressions were downregulated. In addition, RNAi of *JHAMT* decreased JH concentration, delayed the development of reproductive systems, slowed down fat consumption, and delayed the mean mating occurrence time significantly. Conversely, RNAi of *JHEH* resulted in an increased concentration of JH, development of reproductive systems was accelerated, consumption of fat was intensified, and mean mating occurrence time advanced significantly. Taken together, these findings uncovered that JH plays an important role in regulating reproductive diapause in *A. chinensis* and, thus, could provide a theoretical basis for further research on the diapause of *A. chinensis*.

## Introduction

*Aspongopus chinensis* Dallas, 1851, is a Chinese traditional edible and medicinal insect (Guo et al*.*, 2019). The previous studies showed that hemolytic lymph extracted from *A. chinensis* inhibits the activity of cancer cells (Yang et al., 2017; Tan et al., 2019a, Tan et al., 2019b). Therefore, *A. chinensis* is in great demand in the society. However, despite its medicinal value and information about its bioecology being discovered (Wei et al., 2015; Gu et al., 2017), it has a diapause for up to 7 months and reproduces once a year (Wei et al., 2015) which results in insufficient production in the wild resources. To improve the utilization of *A. chinensis* resources, we focused our attention on *A. chinensis* diapause.

Studies have shown that diapause is a developmental arrest that is induced by photoperiod and temperature signals before the unfavorable environmental arrival (Tauber et al., 1986). Insects enter diapause to avoid adverse environments and ensure population continuity (Denlinger 2002). Diapause occurs at the species-specific developmental stage including eggs, larvae, pupas, and adults in insects (Bale and Hayward 2010). Diapause in the adult phase is a reproductive diapause, such as *A. chinensis,* and the main performance is arrested ovarian development and lipid accumulation (Denlinger et al., 2012; Denlinger and Armbruster 2014).

Juvenile hormones (JHs) are considered to be the primary hormones that regulate reproduction in many insects (Jindra et al., 2013; Smykal and Raikhel 2015). The change of juvenile hormone is one of the important factors for the maintenance and termination of diapause in insects. JH was lower during diapause but increased gradually after the diapause termination that was demonstrated in *Drosophila melanogaster*, *Riptortus clavatus*, and *Poecilocoris lewisi* (Herman 1981; Chinzei et al., 1992; Miyawaki et al., 2006). In the absence of JH during diapause, insects mainly exhibit arrested reproductive development, increased lipid accumulation, and higher stress tolerance (Denlinger and Armbruster 2014). In the adult diapause insects, such as Coleoptera, Hemiptera, and Lepidoptera, an injection of exogenous JH or JH analogs (JHAs) promotes reproductive behavior (De Kort 1981; Kort 1990; Kopper et al., 2001; Evenden et al., 2007; Hoffmann et al., 2007; Toyomi et al., 2010; Jindra et al., 2013). However, it remains unknown whether and how does JH terminate diapause in *A. chinensis*.

JH is biosynthesized through the mevalonate pathway. Insects are unable to biosynthesize sterols due to the lack of squalene synthase and lanolin sterol synthase. After farnesyl diphosphate was biosynthesized, JH follows a unique route to biosynthesized (Beenakers et al., 1985). Juvenile hormone acid O-methyl-transferase (JHAMT) is a key enzyme regulated in the JH synthesis pathway and is essential for the normal biosynthesis of JH (Minakuchi et al., 2008). Studies have shown that the use of RNAi to knock down *Schistocerca gregaria JHAMT* will lead to a decrease in the concentration of juvenile hormones and delay the development of the reproductive system (Marchal et al., 2011). In *Blattella germanica*, knockdown with *JHAMT* resulted in a significant decrease in the JH concentration accompanied by a decrease in the expression of the *vitellogenin* gene in the fat body (Dominguez and Maestro 2018). Juvenile hormone epoxide hydrolase (JHEH) has been proved to play an important role in JH degradation (Share et al., 1988; Lassiter et al., 1994; Halarankar et al., 2016). For example, the use of RNAi technology to knockdown with the *JHEH* in *Leptinotarsa decemlineata* larvae will significantly reduce the pupation and molting. In the study of *Coccinella septempunctata* L., it was found that *JHEH* RNAi in diapause adults will increase the concentration of JH, resulting in increasing expression of *vitellogenin* and promoting the development of the reproductive system. In our preliminary study, we conducted a high-throughput transcriptome sequencing analysis of diapause and diapause termination of *A. chinensis* (Wu et al., 2019). The data showed that *JHEH* and *JHAMT* genes have a significant difference in gene expression between diapause and diapause termination. Compared with diapause, *JHAMT* gene expression was significantly upregulated, *JHEH* gene expression was significantly downregulated in diapause termination. Taken together, JH biosynthesis genes *JHAMT* and degradation genes *JHEH* may regulate the concentration of JH together that causes *A. chinensis* to be in diapause state or diapause terminations. However, it remains unknown whether JH participates in reproductive diapause in *A. chinensis*.

In the present study, JH concentration in *A. chinensis* was detected, and results showed that JHIII concentration in diapause individuals was significantly lower than that in diapause termination individuals. Subsequently, the exogenous JHIII was injected into diapause adults of *A. chinensis*. Overall, after JHⅢ injection, the rate of mating increased significantly, development of the reproductive systems accelerated, consumption of fat intensified, the expression of *JHAMT* was upregulated, and *JHEH* and fatty acid synthase (FAS) gene expressions were downregulated. Furthermore, RNAi of *JHAMT* resulted in decreased JH concentration, development of reproductive systems was delayed, the consumption of fat slowed down, and mean mating occurrence time was delayed significantly. Conversely, RNAi of *JHEH* resulted in an increased concentration of JH, development of reproductive systems was accelerated, consumption of fat intensified, and mean mating occurrence time advanced significantly. These findings uncovered that JH plays an important role in regulating reproductive diapause in *A. chinensis* and, thus, could provide a theoretical basis for further research on the diapause of *A. chinensis*.

## Materials and Methods

### Insect Gathering and Rearing

Adults of *A. chinensis* in diapause were collected from Bijie City, Guizhou Province, southwest China. Individuals were kept at 28 ± 1°C with 85 ± 5% humidity under a 16/8 h (light/dark) photoperiod condition (Guo et al., 2019). Mating was used as the standard of diapause termination.

### Juvenile Hormone Ⅲ Determination

The method of extracting juvenile hormone Ⅲ through high-performance liquid chromatography (HPLC) was referenced Hao et al. (2012). The samples of two *A. chinensis* (diapause or diapause termination) were placed in a glass homogenizer, 1 ml methanol and 1 ml ethyl ether was added to the homogenate. After homogenization, 4 ml n-hexane was added. It was then centrifuged at 12,000 r/min for 10 min and supernatant was collected. The precipitation was repeatedly extracted with n-hexane 3 times. The collected liquid samples were dried with a nitrogen blower before placing them into the instrument. The volume was made to 1 ml with the mobile phase. The chromatographic conditions include mobile phase methanol: ether (80: 20), column temperature is 25°C, the flow rate is 0.5 ml/min, sample injected 10 μL, ultraviolet detection wavelength is 218 nm; JHⅢ used isocratic elution, the sample was compared with the prototype of the peak area of quantitative. Each treatment had 3 biological replicates.

### Juvenile Hormone Ⅲ Injecting

The under-wing injection method was used. Exogenous JHIII was diluted with acetone to 5, 10, 50, 100, and 200 ng/μL, acetone as a control. For each adult individual, 2 μL acetone was injected by using a 5 μL microsyringe (Shanghai gaoge industrial and trading co., LTD.). We set five repetitions, each with 10 *A. chinensis* (5 males and 5 females). After injecting, the treated diapause adults were placed in labeled milk tea cups, respectively, in an environment at a temperature of 28 ± 1°C, relative humidity of 85 ± 5%, photoperiod (16 L: 8 D), and the pumpkin (*Cucurbita moschata* Duch) seedlings were used for rearing (Wu et al., 2019). The number of deaths and the termination of diapause were monitored daily.

### dsRNA Treatment

Primers (Table 1) used for dsRNA were designed by Primer 5 program. *JHEH* or *JHAMT* double-stranded RNA (dsRNA) was prepared using the T7 RNA polymerases containing the appropriate gene and injected into *A. chinensis* adult. Adults received 1 μL of dsRNA at a concentration of 400 ng/μL in solution; control insects were injected with *GFP* dsRNA derived from *Aequorea victoria* genes.

**TABLE 1:** Primers for dsRNA synthesis.

Gene names	Primer sequences	bp
dsJHEH-F	taa​tac​gac​tca​cta​tag​ggG​CAG​CAC​TGA​CTA​AAA​GGG​C	434
dsJHEH-R	taa​tac​gac​tca​cta​tag​ggA​ACA​CAG​CAC​AAG​CTG​TTG​G	
dsJHAMT-F	taa​tac​gac​tca​cta​tag​ggA​TTT​TTC​TGC​TGG​GAG​CCT​T	407
dsJHAMT-R	taa​tac​gac​tca​cta​tag​ggT​TGG​ATG​AAT​TGG​GAA​GAG​G	
dsGFP-F	taa​tac​gac​tca​cta​tag​ggG​CCA​ACA​CTT​GTC​ACT​ACT​T	390
dsGFP-R	taa​tac​gac​tca​cta​tag​ggG​GAG​TAT​TTT​GTT​GAT​AAT​GGT​CTG	

### Anatomy of the Reproductive System

Randomly selected *A. chinensis* was treated by dsRNA. The reproductive system was dissected in the precooled 75% alcohol from three females and three males every 7 days until mating to investigate tissue-specific expression profiles under a stereomicroscope (SMZ250, Nikon). Day 0 was the control group. We set three repetitions and carefully measured and recorded the size of the reproductive organs.

### Nile Red Staining of *Aspongopus chinensis* Fat Bodies

Fat bodies were dissected in the precooled phosphate buffer saline, and the adherent fat bodies were carefully removed with forceps as thoroughly as possible under a stereomicroscope (SZM-7045, Nikon). The fat bodies from different time periods (every 7 days) were washed twice with 1× PBS buffer fixed with 4% paraformaldehyde for 30 min at room temperature. It was then washed twice with 1× PBS buffer. The fat bodies were then incubated in Nile Red solution (1 mg/ml Nile Red was diluted to 1 ug/mL by 0.1% Triton X-100) for 90 min at room temperature to stain the lipid droplets. Then it was washed twice with 1× PBS buffer. The cell nuclei were stained with 1 μg/ml DAPI (Sigma, D8417, United States) for an additional 15 min. Finally, it was washed twice with 1× PBS buffer. Fat body cell images were captured using a Nikon bright-field inverted light microscope (Tokyo, Japan). Day 0 was the control group. We set three repetitions, each with six *A. chinensis* (three males and three females).

### Detection of Triglyceride Content

The total TG levels were determined by the liquid triglycerides (GPO-PAP) method (Liu et al., 2016) using the Triglycerides Assay Kit (Nanjing Jiancheng Institute, China) according to the manufacturer’s instructions. After accurately weighing the fat body, we added anhydrous ethanol at a weight (g) to volume (ml) ratio of 1: 9. Mechanical homogenization was conducted under ice bath condition. Then, after centrifugation at 2,500 rpm for 10 min, the supernatant was taken; 2.5 μL of the supernatant and 250 μL reaction solution were added to a 96-well plate and incubated at 37°C for 10 min. The absorbance was measured at 510 nm using an automated microplate reader (Bio-Rad, Hercules, CA, United States). Three independent biological replicates and five technical replicates were performed for every treatment. The formula used for calculation was as follows.

### Quantitative Real-Time PCR

Total RNAs from whole bodies were isolated using RNAeasy™ Animal RNA isolation Kit with Spin Column (Shanghai, Beyotime) according to the manufacturer’s instructions. RNA concentration was determined using a spectrophotometer (ND-2000, United States, Thermo Scientific). To avoid genomic DNA contamination, total RNA was treated with RNase-free DNase water (Pekin, Solarbio). The first-strand cDNA was synthesized using a SuperScript double-stranded cDNA synthesis kit according to the manufacturer’s instructions. Primers (Table 2) used for qPCR were designed by the Primer 5 program. qPCR was performed with a T100TM PCR instrument (T100TM, United States, Bio-Rad) using the iTaqTM Universal SYBR®Green Supermix under the following reaction conditions: 95°C for 10 min; 40 cycles of 94°C for 30 s, 53°C for 30 s, and 72°C for 20 s. The stable reference gene beta-actin of *A. chinensis* was used for normalization (Wu 2019). All qPCR reactions were carried out in three biological replicates, and three technical replicates were performed for each sample. The relative expression levels were calculated using the 2^−ΔΔCT^ method (Livak and Schmittgen 2001).

**TABLE 2:** qPCR primers.

ID	Forward primer	Reverse primer	bp
*JHEH*	TTC​ATC​AGG​TGG​GTG​CTA​AGA	CCA​GTC​TTG​CCA​TAA​GTT​CAT​T	146
*JHAMT*	TCC​TCT​TCC​CAA​TTC​ATC​CAA​A	ACC​TCA​CAA​CTT​CCC​TTC​TAA​C	172
*FAS*	TGT​AGA​TGT​AGT​CGT​TCA​GAG​G	GTC​GTA​AGG​AGC​GGA​GTC​T	106

### Data Analysis

Results were presented as mean ± SE (standard error) based on at least three independent biological replications. Differences between the two groups were analyzed by an independent *t*-test. One-way ANOVA followed by Duncan’s multiple comparison was used for the comparison among more than two different conditions. *p* values less than 0.05 (*), 0.01 (**), or 0.001 (***) were considered to be statistically significant. All data were subjected to statistical analysis using the SPSS software (version 22; SPSS Inc., Chicago, IL, United States). Graphical representations were performed using Excel 2007 software.

## Results

### JHIII in Diapause Was Lower Than That in Diapause Termination

**FIGURE 1:**
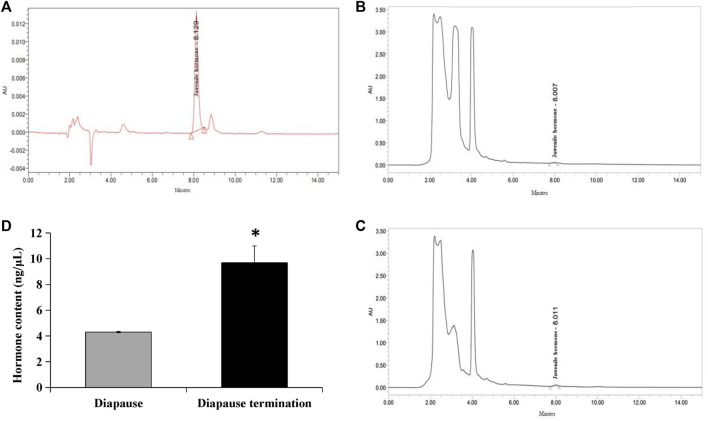
Determination of JHⅢ in diapause and diapause termination adult individuals. **(A)** Chromatogram of JHIII standard sample. **(B)** Chromatogram of JHIII in diapause of *A. chinensis.* **(C)** Chromatogram of JHIII in diapause termination of *A. chinensis.* **(D)** Quantification of JHIII content in diapause and diapause termination. *, *p* < 0.05 (independent *t*-test).

High-performance liquid chromatography was used to detect juvenile hormones. The result indicated that the UV absorption peak of standard JHIII was at 218 nm, and the retention time was 8 min approximately (Figure 1A). Subsequently, the JHⅢ of *A. chinensis* in diapause and diapause termination were detected. A small peak appeared at the time close to the standard sample both in diapause (Figure 1B) and diapause termination samples (Figure 1C). However, the peak in the chromatogram of diapause termination was higher than that in diapause. The JHIII content was quantified according to the peak areas of samples and standards, and the data showed that JHIII concentration in diapause (4.3016 ± 0.0449 ng/μL) was significantly lower than that in diapause termination (9.6873 ± 1.6157 ng/μL) (Figure 1D). It implies that the increase in JHIII content in *A. chinensis* was involved in terminating diapause.

### Exogenous JHIII Injection Affects Reproductive Diapause

To further explore the relationship between JH and *A. chinensis* diapause, the exogenous JHIII injecting was employed. The survival rate of *A. chinensis* was above 90% after injecting with different JHIII concentrations (Supplementary Table S1). In addition, the rate of mating was counted at day 30. The results showed that JHⅢ injection could increase the rate of mating significantly, and 50 ng/μL JHIII injection had the highest mating rate (91.600% ± 0.038%) (Table 3).

**TABLE 3:** Mating rates after JHIII injection.

Concentration of JHⅢ (ng/μL)	Mating (%)
CK	0.000 ± 0.000^c^
5	0.000 ± 0.000^c^
10	21.700 ± 0.100^b^
50	91.600 ± 0.038^a^
100	39.330 ± 0.785^b^
200	21.880 ± 0.094^b^

The data in the table are mean ± SE. The different lowercase letters in the upper right corner represent a significant difference at p < 0.05 (one-way ANOVA followed by Duncan’s multiple comparison).

**FIGURE 2:**
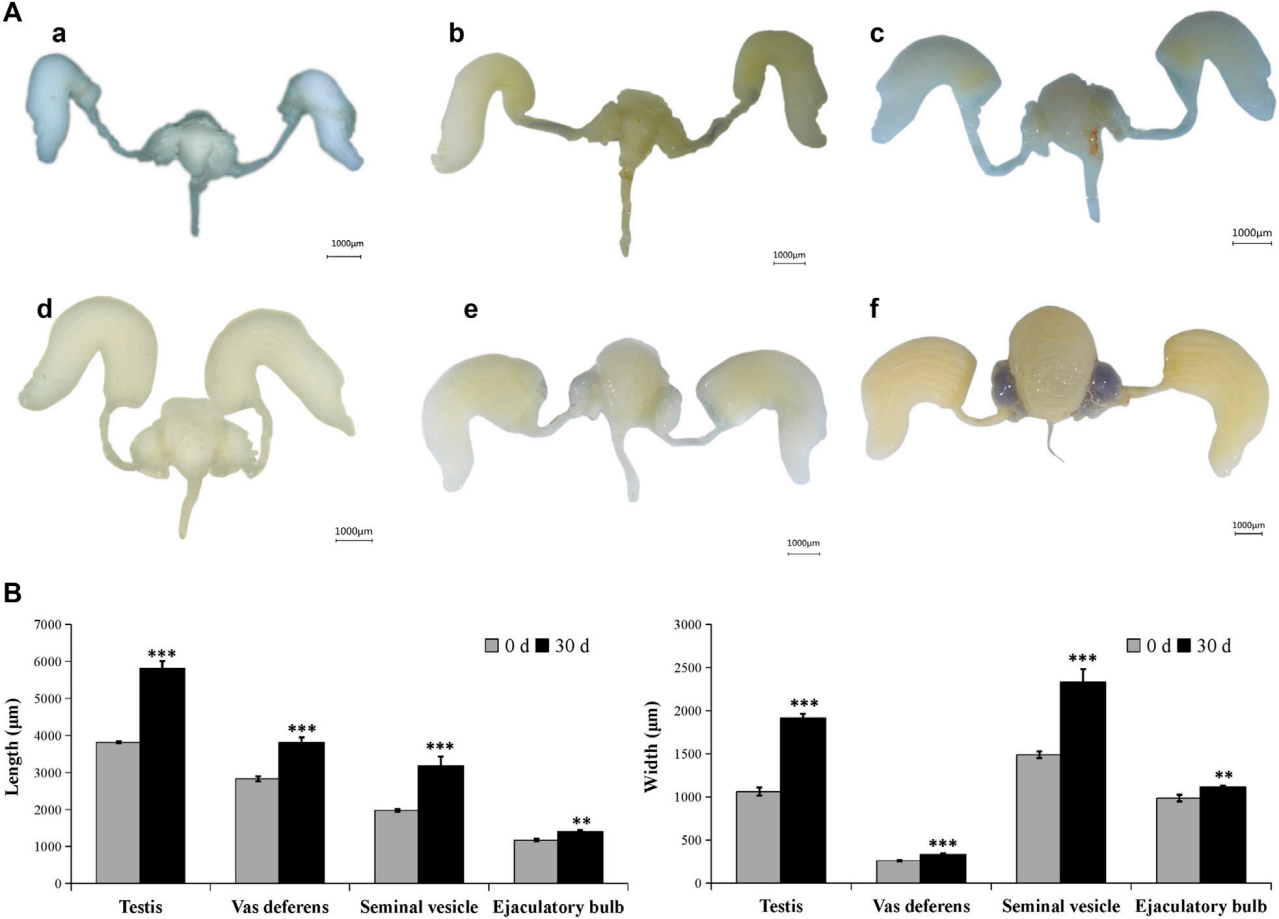
Development pattern of male reproductive system after JHIII injection. **(A)** Photograph of male reproductive system at different day after JHIII injection. a, 0th day; b, 7th day; c, 14th day; d, 21st day; e, 28th day; f, 30th day. **(B)** Length and width of male reproductive structures (testis, vas deferens, seminal vesicle, ejaculatory bulb). **, *p* < 0.01; ***, *p* < 0.001 (independent *t*-test).

**FIGURE 3:**
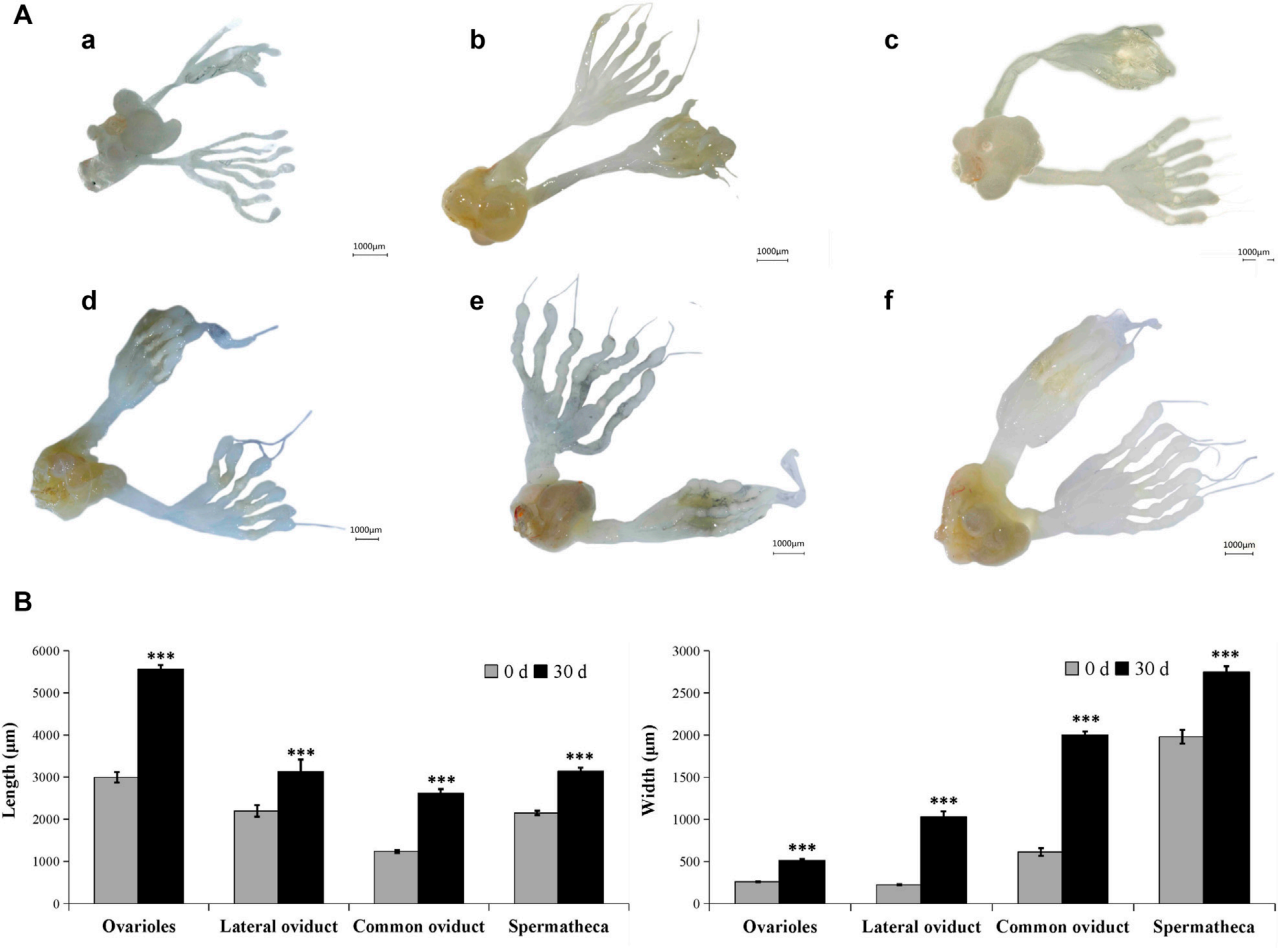
Development pattern of female reproductive system after JHIII injection. **(A)** Photograph of female reproductive system at different day after JHIII injection. a, 0th day; b, 7th day; c, 14th day; d, 21st day; e, 28th day; f, 30th day. **(B)** Length and width of female reproductive structures (ovarioles, lateral oviducts, common oviduct, spermatheca). ***, *p* < 0.001 (independent *t*-test).

In the male adult, the reproductive system is mainly composed of six parts: a pair of testis (which is divided into seven testicular follicles), a pair of vas deferens, a seminal vesicle, an ejaculatory bulb, an ejaculatory duct, and a pair of accessory glands (Supplementary Figure S1). Correspondingly, in the female adult of *A. Chinensis*, the reproductive system is mainly composed of a pair of ovaries (which divided into seven ovarioles and with a terminal filament at the apex of each ovariole), a pair of the lateral oviduct, a common oviduct, and a pear-shaped spermatheca, with no accessory glands (Supplementary Figure S2). However, after injection of exogenous JHIII, the male (Figure 2) and female (Figure 3) reproductive organs of A. *Chinensis* developed rapidly to maturity in about 30 days, and the length and width of reproductive system structures (testis, vas deferens, seminal vesicle, ejaculatory bulb in male; ovarioles, lateral oviducts, common oviduct, spermatheca in female) increased significantly.

**FIGURE 4:**
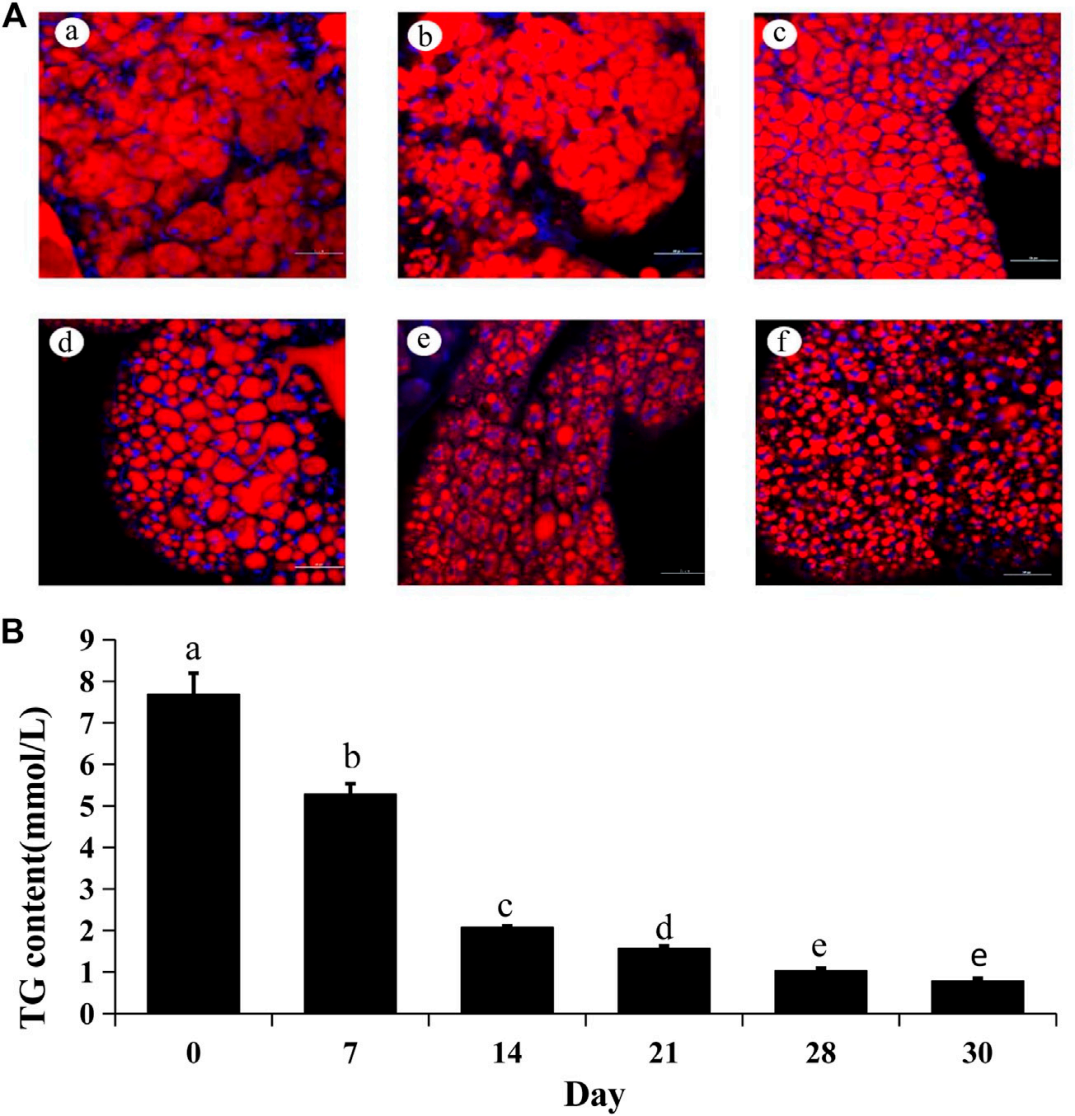
Lipid changes after JHIII injection in the fat body of *A. chinensis*. **(A)** Nile Red staining of fat body after JHIII injection. a, 0th day; b, 7th day; c, 14th day; d, 21st day; e, 28th day; f, 30th day. Red signal indicates the lipid droplets stained by Nile Red, and blue signal indicates the nucleus stained by DAPI. **(B)** Triglyceride (TG) contents. The different lowercase letters above bar are significant difference at *p* < 0.05 (one-way ANOVA followed by Duncan’s multiple comparison).

The lipid droplets were consumed as energy for reproductive development after diapause termination. Thus, Nile Red staining was used to detect the change of lipid droplets after JHIII injection. In the beginning, the lipid droplets filled the whole fat body. After JHIII injection, the stored lipid droplets in *A. chinensis* were reduced over time (Figure 4A). In addition, the triglycerides levels were reduced after JHIII injection which is consistent with the results of Nile Red staining (Figure 4B).

**FIGURE 5:**
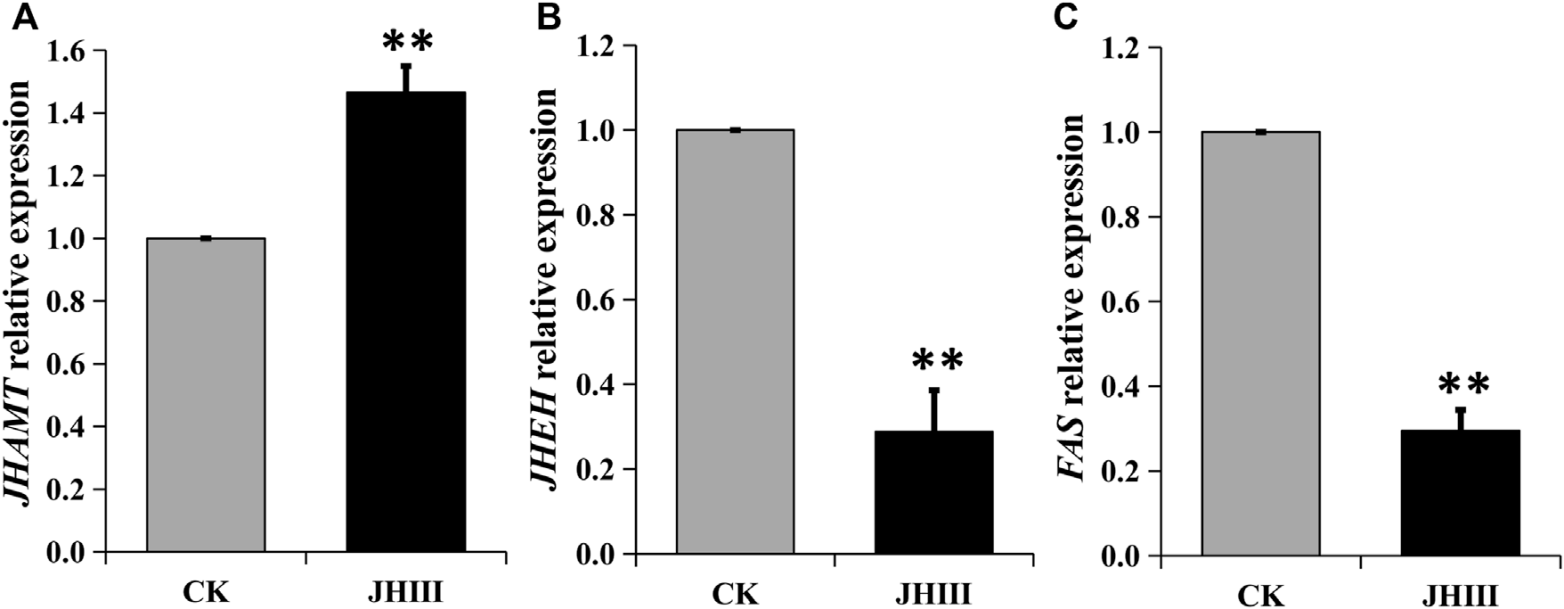
Relative expression of *JHAMT* **(A)**, *JHEH* **(B)**, *FAS* **(C)** genes after JHIII injection. **, *p* < 0.01 (independent *t*-test).

After the exogenous JHIII was injected into diapause adults of *A. chinensis*, the relative expressions of *JHEH*, *JHAMT*, and *FAS* were measured by qPCR. The data showed that the expression of the *JHAMT* was upregulated significantly. Whereas the expressions of *JHEH* and *FAS* were downregulated significantly (Figure 5).

The results given above demonstrated that JH could promote reproductive development but reduce lipid storage suggesting that JH has opposite effects on reproductive diapause.

### Effects of *JHEH* and *JHAMT* RNAi on Reproductive Development

**FIGURE 6:**
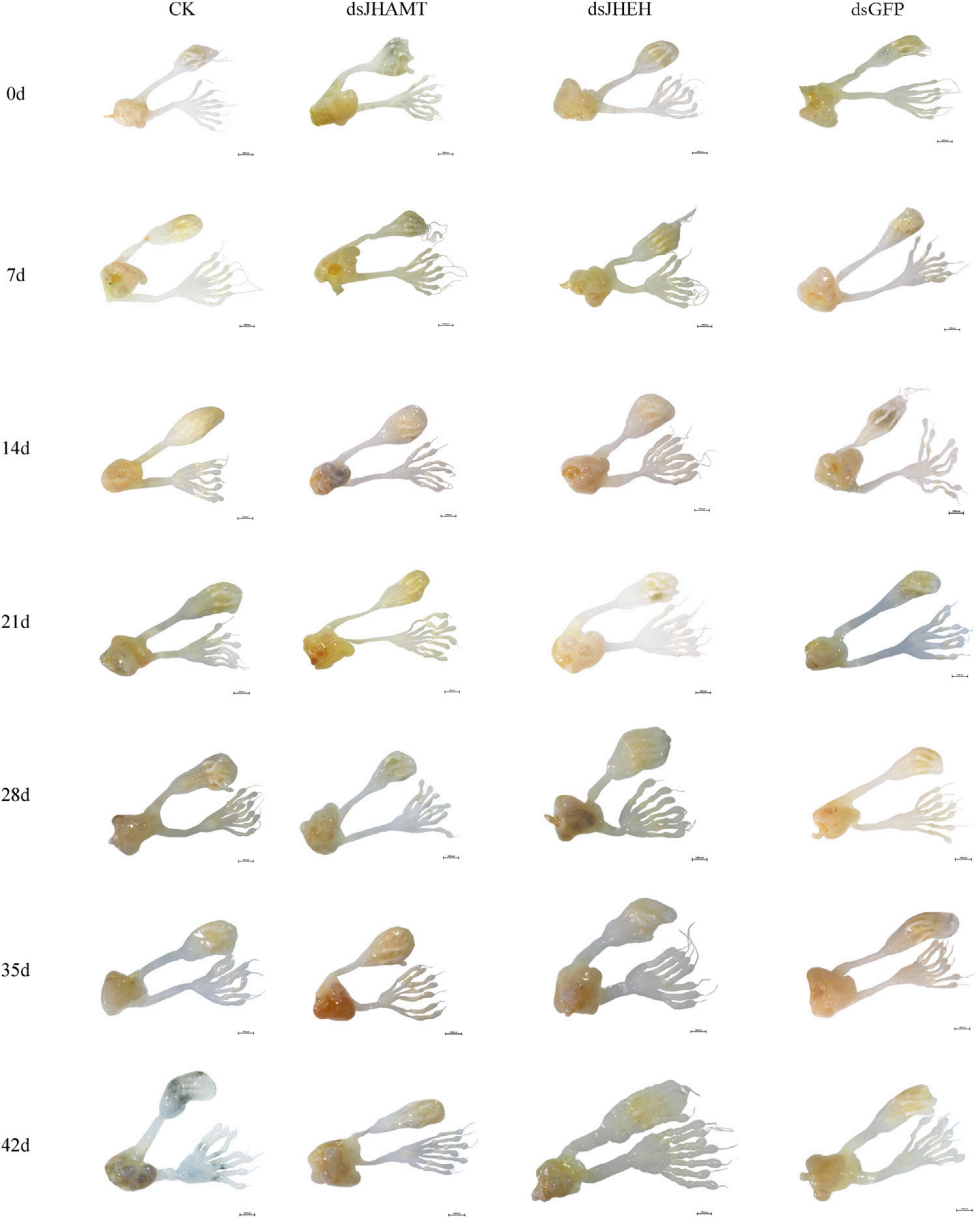
Development pattern of male reproductive system after *A. chinensis* injected with dsRNA.

**FIGURE 7:**
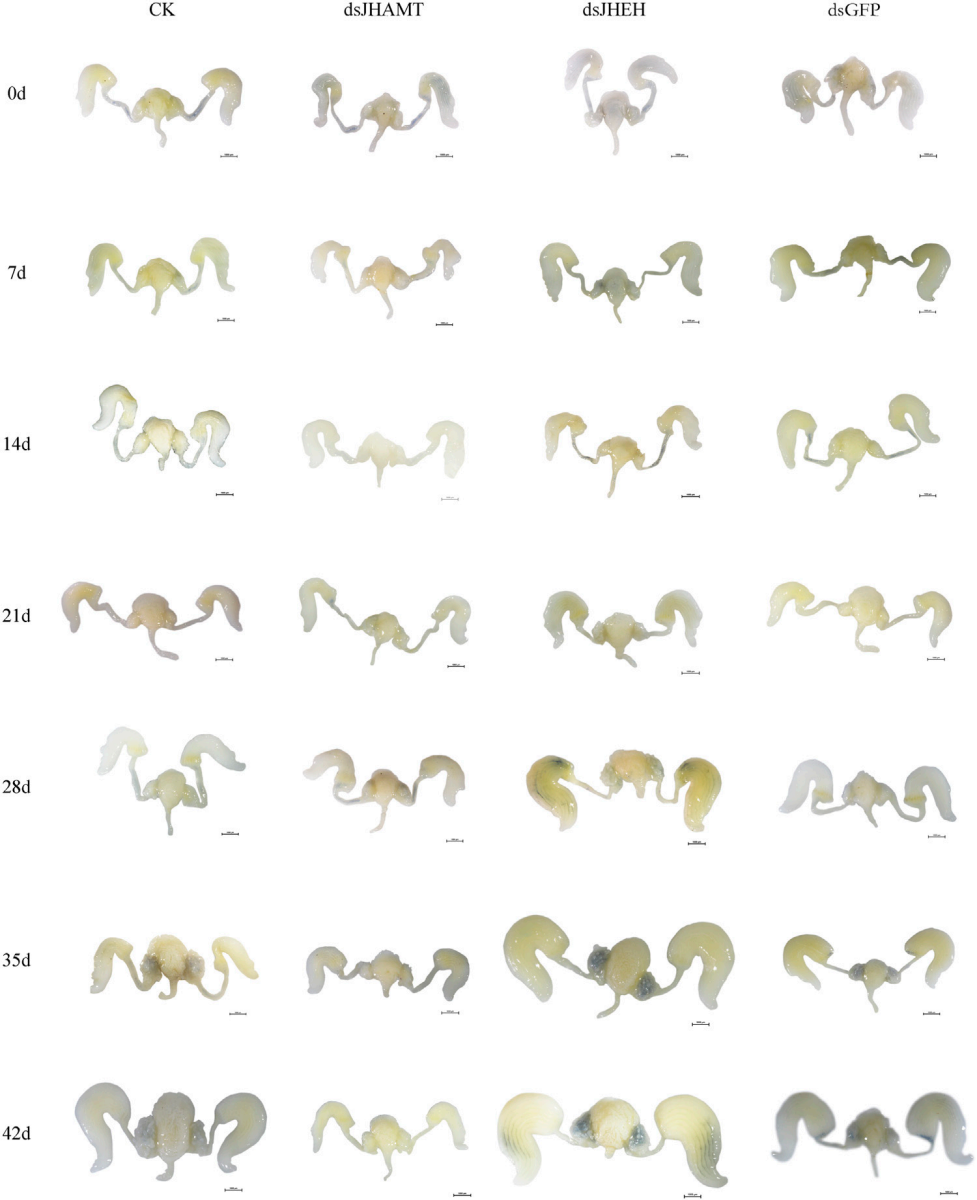
Development pattern of female reproductive system after *A. chinensis* injected with dsRNA.

Firstly, we synthesized the dsRNA of *JHEH* and *JHAMT* genes, and the expressions of *JHEH* and *JHAMT* in *A. chinensis* were significantly inhibited after injection of dsRNA (Supplementary Figure S3). The inhibition rate was more than 80%, indicating that the interference effect is better. Next, the anatomy of the reproductive system of the *A. chinensis* after dsRNA injection is shown in Figures 6, 7. Compared with the control group (CK or dsGFP), the male or female reproductive system of the dsJHAMT experimental group developed significantly slower, while the dsJHEH experimental group developed faster. With the passage of time, the testis, vas deferens, seminal vesicles and ejaculatory bulb gradually developed in the ds*JHEH* group developed and matured earlier, while the dsJHAMT group developed more slowly (Supplementary Figure S4). The ovarioles, lateral oviduct, spermatheca, and common oviduct in each experimental group developed gradually over time, and the dsJHAMT group developed slowly at 0, 7, 14, 21, 28, 35, and 42 days, while the dsJHEH group developed on the 42nd day (Supplementary Figure S5).

**FIGURE 8:**
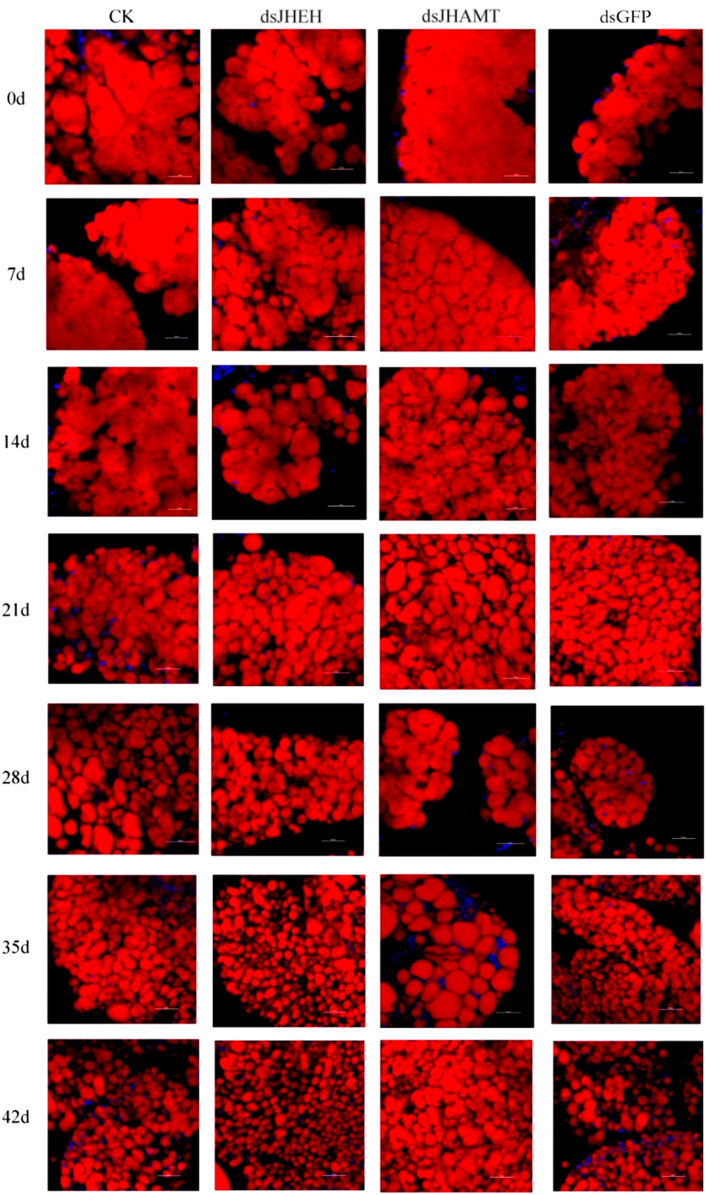
Lipid change in the fat body after injection of dsRNA in *A. chinensis*. Red signal indicates the lipid droplets stained by Nile Red, and blue signal indicates the nucleus stained by DAPI.

**FIGURE 9:**
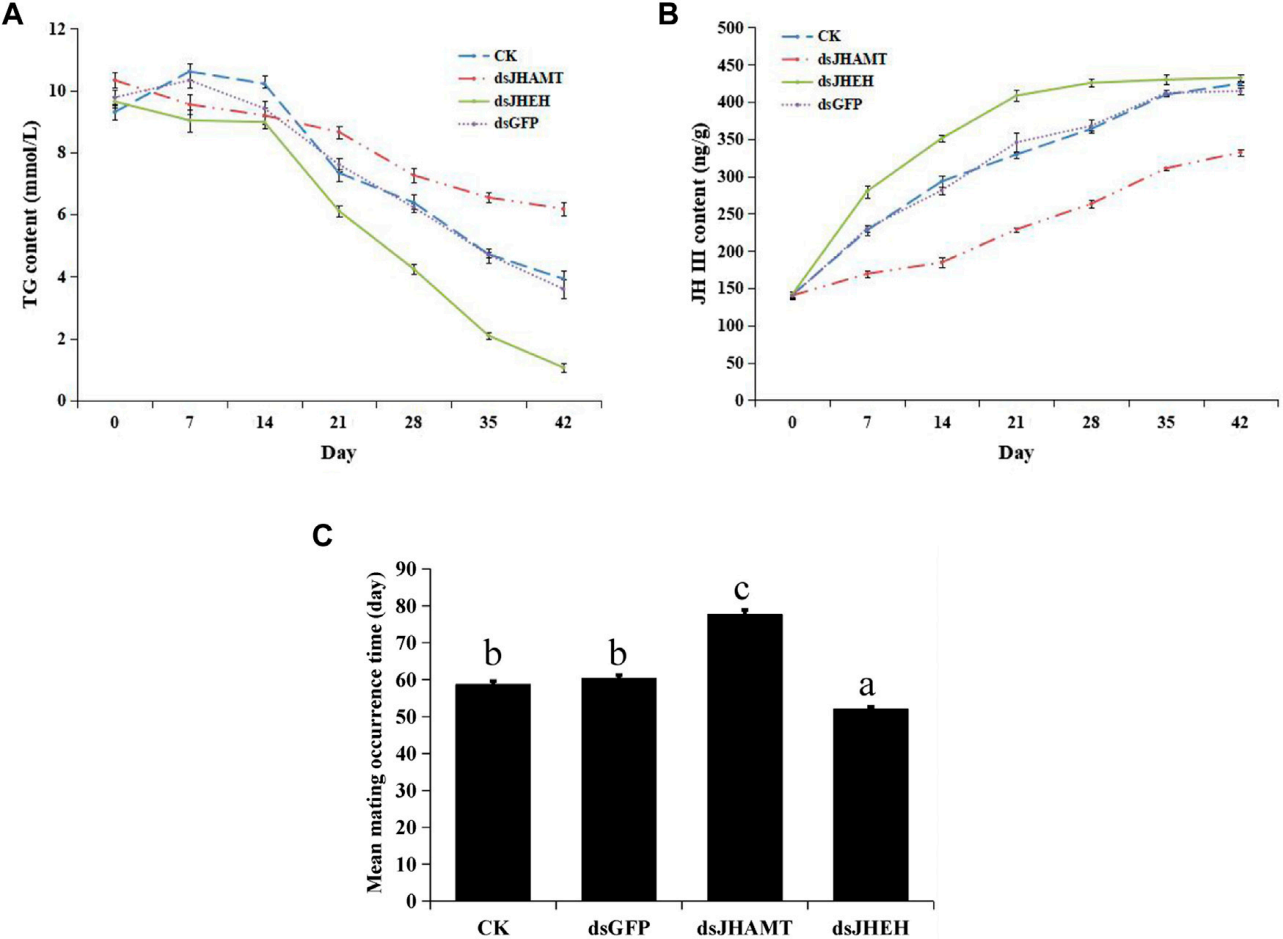
Triglyceride content **(A)**, juvenile hormone concentration **(B)** and mean mating occurrence time **(C)** after dsRNA injection. The different lowercase letters above bar are significant difference at *p* < 0.05 (one-way ANOVA followed by Duncan’s multiple comparison).

The results of Nile Red staining showed that the fat body cells in the early stage of diapause were full of lipids, and the fat was gradually consumed over time. Compared with the CK and dsGFP group, the fat consumption of dsJHEH was significantly higher, while the fat consumption of dsJHAMT was not obvious (Figure 8). Compared with the control group (dsGFP) and the blank group (CK), the consumption rate of triglycerides in the dsJHAMT group was relatively slow, while the consumption of triglycerides in the dsJHEH group was intensified (Figure 9A), which was consistent with the results of Nile Red staining.

We calculated the concentration of juvenile hormone according to the standard curve (Supplementary Figure S6). Juvenile hormones in diapause *A. chinensis* are a process of accumulation (CK group in Figure 9B). In the dsGFP group, the concentration of juvenile hormone increased from 140.04 ng/g to 414.38 ng/g with time. After injection of dsJHEH, the degradation of juvenile hormone was inhibited, and the growth rate of juvenile hormone concentration was significantly higher than that of other groups. Compared with dsGFP, after injection of dsJHAMT, the synthesis of juvenile hormone was significantly inhibited, and the growth rate of juvenile hormone was slowed down (Figure 9B and Supplementary Table S2).

At last, the mean mating occurrence time after dsRNA injection was calculated. The results showed that the mean mating occurrence times of the CK and dsGFP groups were at 58.67 ± 0.88 and 60.33 ± 0.88 days, respectively. The injection of dsJHAMT was at 77.67 ± 1.20 days, which was delayed significantly compared with other groups, while the dsJHEH injection group was at 52.00 ± 0.58 days, and their mating occurrence time was significantly earlier (Figure 9C).

## Discussion

Diapause can occur in any period in insects including eggs, larvae, pupae, and adults. It is well known that hormones, such as diapause hormone, ecdysteroids, and JH, control insect diapause (Denlinger 2002). The obligate diapause of *A. chinensis* occurred in adults complied by reproductive arrested. Most of the previous works on adult diapause focused on JH, and the results supported that JH plays a major role in regulating adult diapause in insects (Denlinger et al., 2012). In the present study, we found JH concentration was lower in diapause adults than in diapause determination adults. JH application can stimulate reproductive maturation and terminated diapause. These results suggested JH is involved in *A. chinensis* diapause which is consistent with the adult diapause study in other insects. However, JH is not the exclusive cause of diapause induction and maintenance. For example, in *D. melanogaster*, the ovaries produce a low level of ecdysteroids during diapause but a high level in terminating diapause (Richard et al., 1998), and 20-hydroxyecdysone (20E) injection can terminate diapause in a dose-dependent manner (Richard et al., 2001). Similar results were found in *Colaphellus bowringi* (Guo et al., 2021). In addition, a study in *L. decemlineata* showed that injection of a combination of 20E and JH are much more effective in breaking diapause than only JH injection (Lefevere 1989). The evidences above imply that both JH and ecdysteroid have been implicated in the regulation of diapause. But in *A. chinensis*, other hormones, such as ecdysteroid, are involved in diapause regulation should be further studied.

JHAMT and JHEH are key enzymes in JH synthesis and degradation, respectively. RNAi of *JHAMT* decreased the JH concentration, and *JHEH* knockdown increased the concentration of JH. Especially, when exogenous JH injection resulted in increasing *JHAMT* gene expression and downregulating *JHEH* gene expression. This performance uncovers that JHAMT or JHEH with juvenile hormone exits feedback regulation. Nevertheless, there are many genes in the decision of JH concentration except for JHAMT and JHEH, and other genes involved in diapause of *A. chinensis* need further study. On the other side, despite we found JH to play an important role in the regulation of *A. chinensis* adult diapause, the downstream response mechanism remains a mystery. However, some cell signaling pathways, such as insulin signaling, have been confirmed to regulate insect diapause (Hahn and Denlinger 2011). In the study of *Culex pipens*, the addition of JH to diapausing individuals reduces FoxO (a key intracellular effector in insulin signaling) levels in the fat body (Sim and Denlinger 2013). Moreover, in *A. chinensis*, the transcriptome analysis discovered that the FoxO gene expression was a significant difference between diapause adults and diapause termination adults (Wu et al., 2019). Therefore, insulin signaling may be downstream of JH for regulating adult diapause in *A. chinensis*.

It is known that lipids, especially triacylglyceride, are the most important energy reserve in most diapausing insects. Diapause individuals have greater triacylglyceride accumulation than development individuals in some species, and increased triacylglyceride storage is thought to be an important factor for mitigating the metabolic demands of diapause (Hahn and Denlinger 2007). Further, when lipid metabolic rate is high, the duration of diapause is shorter, and when the metabolic rate is low, the diapause duration is longer. Presumably, these supported that the amount of stores or rate of utilization of lipid adjusted the duration of diapause (Hahn and Denlinger 2011). In *A. chinensis*, we found the lipid droplets fill the whole fat body in the earlier diapause, and the consumption of lipid was accomplished with diapause maintenance. When exogenous JH application was done in diapause *A. chinensis*, lipid consumption was increased. The results suggest lipid or triacylglyceride may play an important role in diapause maintenance, and JH targeted the downstream lipid for elevating the metabolic rate resulting in a shorter diapause. However, another previous study has proposed that lipid accumulation in *Polistes* wasps produces JH suppression which is a prerequisite for diapause (Hunt et al., 2007). From this, JH and lipid regulated adults’ diapause may play a role in reciprocation and intricacy, which will be an open question.

The central feature of adult diapause is reproductive arrest. Most previous studies were focused on females, and oocyte development arrest was characterized for the diapause. However, there are few studies conducted on reproductive diapause in male insects (Denlinger et al., 2012). Here, we focus on the reproductive diapause in female adults and male adults equally, and the reproductive system characteristics of diapause males were observed in detail. Furthermore, the previous view states that the status of the testes may not be a consistent reliable indicator of diapause in males. Thus, in males, the most conspicuous feature was their failure to mate with receptive females which were used to determine diapause in a few studies (Pener 1992). Whereas, in our findings, similar to females, the developmental stages of the male reproductive organ are stable and observable either in JH application or RNAi injection. These results indicated that male reproductive organs (including testes) can be used for diapause monitoring in *A. chinensis*.

In summary, our study found that JH was lower in diapause adults than that in diapause termination adults in *A. chinensis*. Exogenous JH application can terminate diapause, promoted the development of the reproductive system both in males and females, and accelerated lipid consumption. For another, the disruption of *JHEH* and *JHAMT* modulated the concentration of JH, promoted or inhibited the reproductive system development and lipid consumption. This funding uncovered that the JH is an important factor in regulating adult diapause in *A. chinensis* and, thus, could provide a basis for further research.

## Data Availability

The original contributions presented in the study are included in the article/Supplementary Material, further inquiries can be directed to the corresponding authors.

## Supplementary Material

Supplementary Figure S1 Internal reproductive organs of a sexually mature male of *A. chinensis*, dorsal view **(A)** and ventral view **(B)**. AG, accessory gland; ED, ejaculatory duct; EB, ejaculatory bulb; SV, seminal vesicle; T, testis; VD, vas deferens.

Supplementary Figure S2 Reproductive organs of a sexually mature female of *A. chinensis*. CO, common oviduct; LO, lateral oviduct; Ov, ovary; Ovl, ovariole; S, spermatheca; TF, terminal filament.

Supplementary Figure S3 Relative expression of JHEH **(A)** and JHAMT **(B)** in *A. chinensis* after RNAi. **, *p* < 0.01 (independent *t*-test).

Supplementary Figure S4 Males reproductive system development after RNAi in *A. chinensis*. Average length and width measurements of testis **(A, B)**, vas deferens **(C, D)**, seminal vesicle **(E, F)** and ejaculatory bulb **(G, H)**. The different lowercase letters above bar are significant difference at *p* < 0.05 (one-way ANOVA followed by Duncan’s multiple comparison).

Supplementary Figure S5 Female reproductive system development after RNAi in *A. chinensis*. Average length and width measurements of ovarioles **(A, B)**, lateral oviduct **(C, D)**, spermatheca **(E, F)** and common oviduct **(G, H)**. The different lowercase letters above bar are significant difference at *p* < 0.05 (one-way ANOVA followed by Duncan’s multiple comparison).

Supplementary Figure S6 Standard curve of juvenile hormone.

Supplementary Table S1 Survival of diapause *A. chinensis* injected with JHIII for 48 h.

Supplementary Table S2 Juvenile hormone concentration after dsRNA injection.
