# Supplementary material for: Juvenile Hormone Is an Important Factor in Regulating Aspongopus chinensis Dallas Diapause
Source: Front Physiol. 2022 May 9;13:873580. doi: 10.3389/fphys.2022.873580 (PMC9124767; doi:10.3389/fphys.2022.873580)
Supplement: Supplementary file 1 [file DataSheet1.pdf]

Supplementary files

Table S1 Survival of diapause *A. chinensis* injected with JHIII for 48 h.

Concentration of JHIII (ng/μL)	Survival rates (%)
CK	96.000±0.024[a]
5	96.000±0.024[a]
10	96.000±0.024[a]
50	96.000±0.024[a]
100	92.000±0.037[a]
200	94.000±0.040[a]

Note: The data in the table are mean±SE. The different lowercase letters in the same column are significantly different at $P<0.05$ (One-way ANOVA followed by Duncan's).

Table S2 Juvenile hormone concentration after dsRNA injection.

Day	CK（ng/g）	dsJHAMT（ng/g）	dsJHEH（ng/g）	dsGFP（ng/g）
0	140.98±4.71[a]	140.16±5.45[a]	141.69±3.35[a]	140.04±1.45[a]
7	227.39±6.56[b]	169.30±4.85[a]	279.85±8.07[c]	230.10±4.31[b]
14	293.24±7.29[b]	184.53±7.13[a]	351.01±4.65[c]	281.22±5.55[b]
21	329.24±4.58[b]	228.66±2.76[a]	408.31±7.56[c]	345.48±13.57[b]
28	363.61±2.06[b]	263.06±5.21[a]	425.46±4.93[c]	367.69±4.73[b]
35	409.63±1.51[b]	310.71±2.11[a]	429.68±6.46[b]	411.92±4.73[b]
42	424.38±1.74[b]	332.11±4.20[a]	432.28±3.96[b]	414.38±4.85[b]

Note: The data in the table are mean±SE. The different lowercase letters in the upper right corner represents significant difference at $P<0.05$ (One-way ANOVA followed by Duncan')

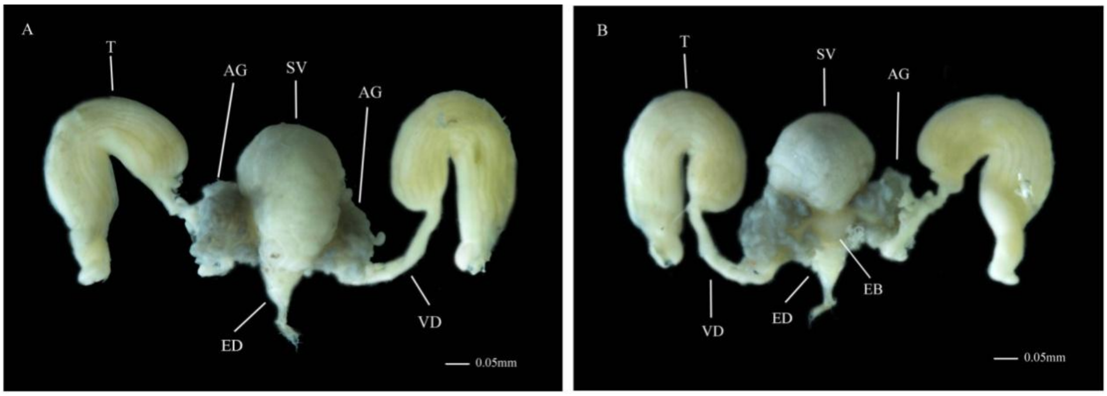

Fig. S1 Internal reproductive organs of a sexually mature male of *A. chinensis*, dorsal view (A) and ventral view (B). AG, accessory gland; ED, ejaculatory duct; EB, ejaculatory bulb; SV, seminal vesicle; T, testis; VD, vas deferens.

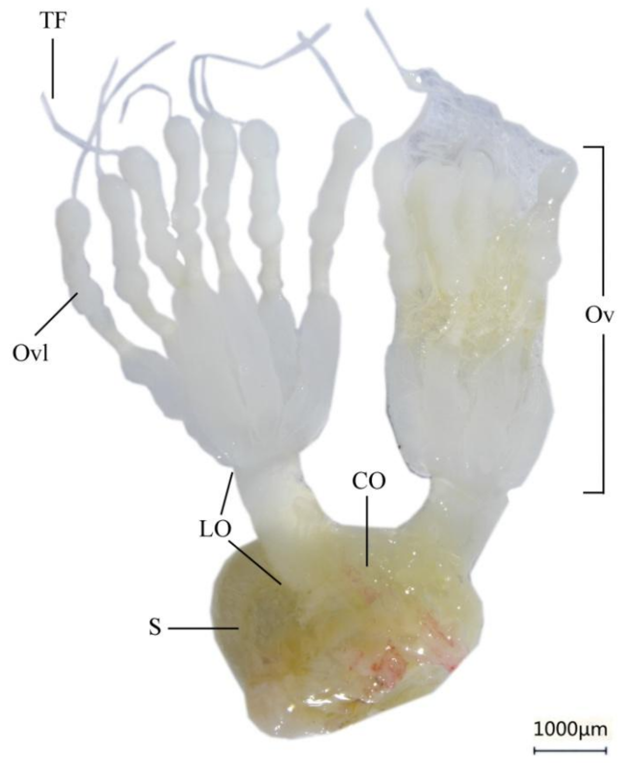

Fig. S2 Reproductive organs of a sexually mature female of *A. chinensis*. CO, common oviduct; LO, lateral oviduct; Ov, ovary; Ovl, ovariole; S, spermatheca; TF, terminal filament.

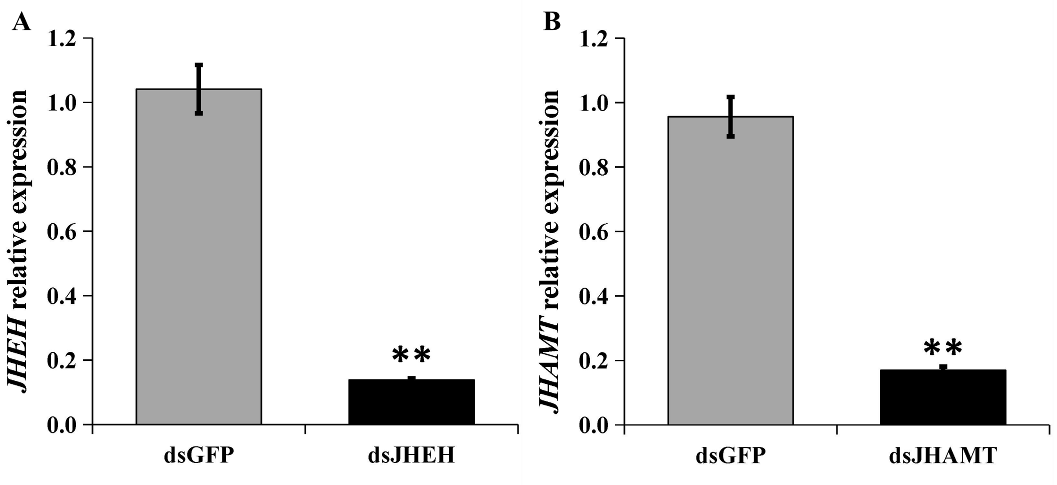

Fig. S3 Relative expression of *JHEH* (A) and *JHAMT* (B) in *A. chinensis* after RNAi. **, $P<0.01$ (independent t-test).

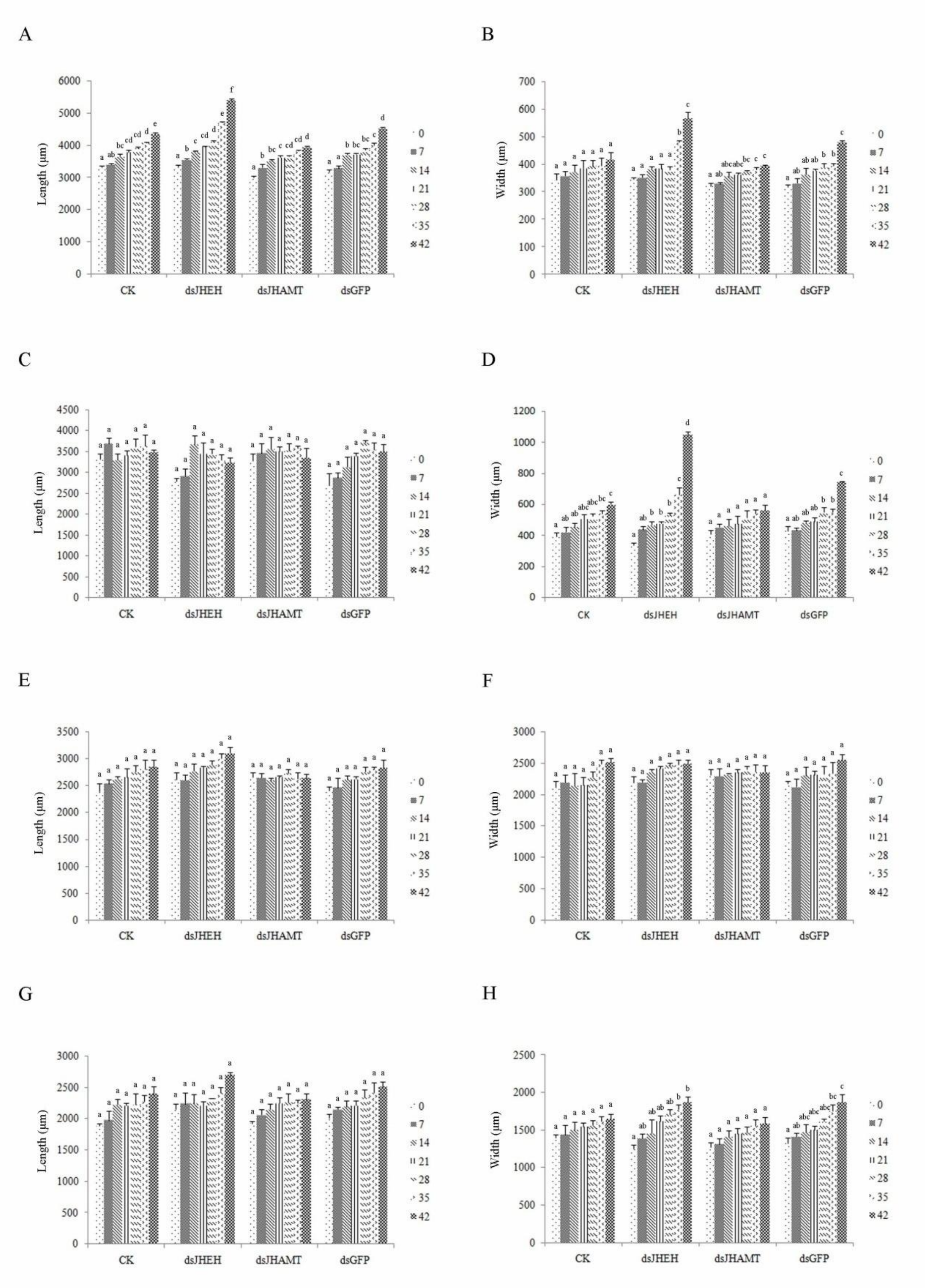

Fig. S4 Males reproductive system development after RNAi in *A. chinensis*. Average length and width measurements of testis (A, B), vas deferens (C, D), seminal vesicle (E, F) and ejaculatory bulb (G, H). The different lowercase letters above bar are significant difference at $P<0.05$ (One-way ANOVA followed by Duncan's).

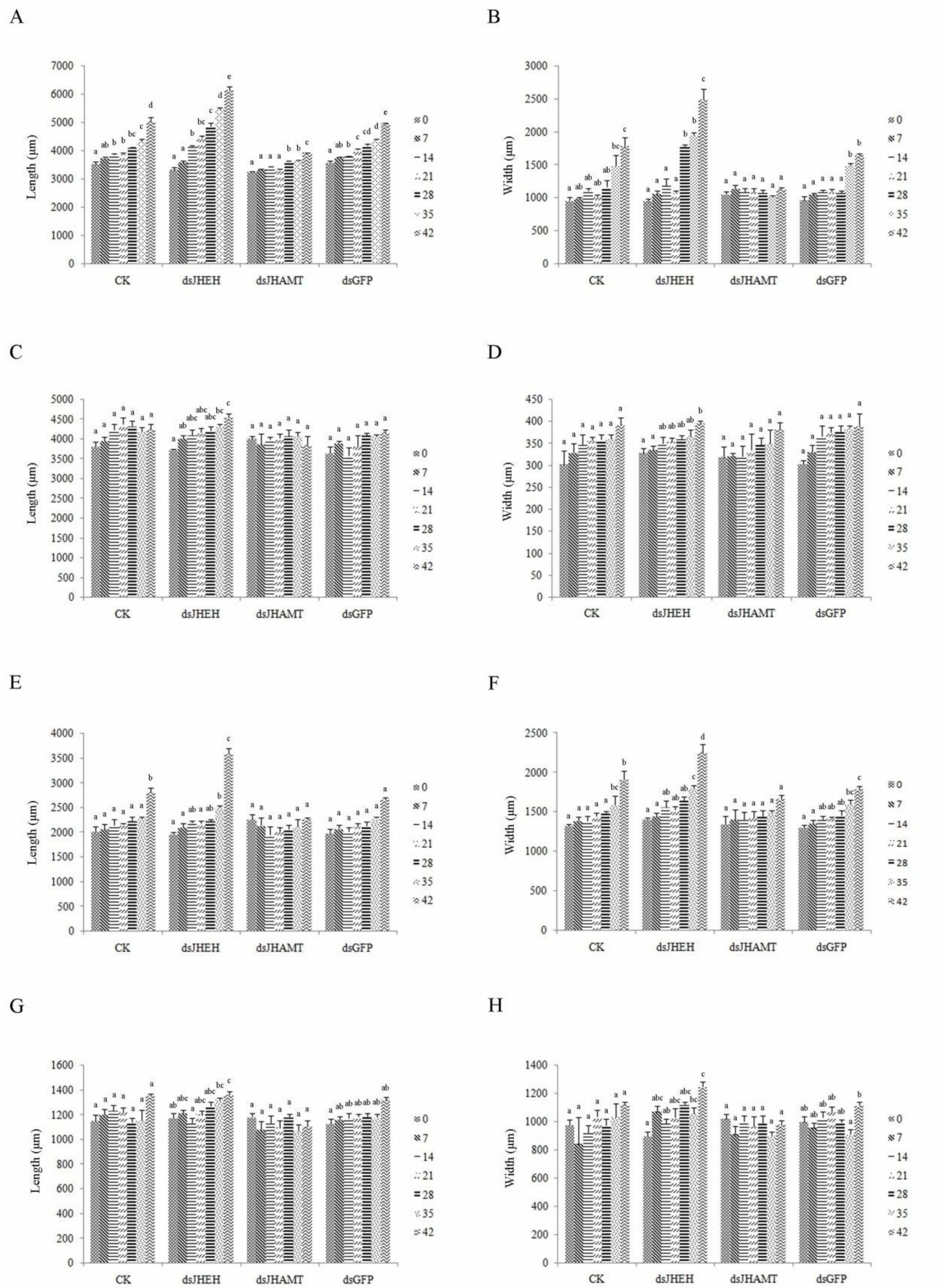

Fig. S5 Female reproductive system development after RNAi in *A. chinensis*. Average length and width measurements of ovarioles (A, B), lateral oviduct (C, D), spermatheca (E, F) and common oviduct (G, H). The different lowercase letters above bar are significant difference at $P < 0.05$ (One-way ANOVA followed by Duncan's).

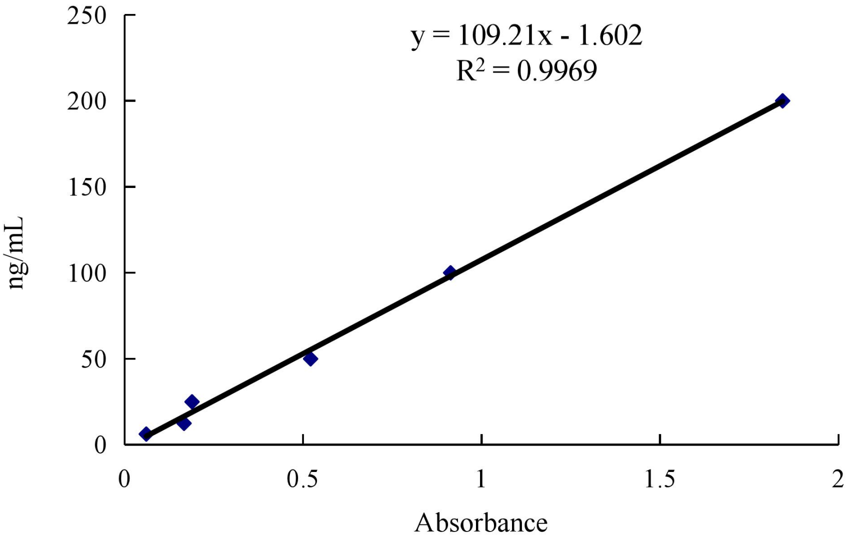

Fig. S6 Standard curve of juvenile hormone.
